# Green extraction of bioactive components from carrot industry waste and evaluation of spent residue as an energy source

**DOI:** 10.1038/s41598-022-20971-5

**Published:** 2022-10-05

**Authors:** Prabhjot Kaur, Jayasankar Subramanian, Ashutosh Singh

**Affiliations:** 1 School of Engineering, University of Guelph, Guelph, ON N1G2W1 Canada; 2 Plant Agriculture, University of Guelph, Guelph, ON Canada

**Keywords:** Isolation, separation and purification, Energy, Green chemistry

## Abstract

Carrot processing industries produce 25–30% of waste in the form of carrot rejects, peels, and pomace which contain a large amount of high-value bioactive components. Green extraction of the bioactive components from carrot rejects with green solvents using closed-vessel energy-intensive microwave-assisted extraction was the objective of this work. In this work, three experimental studies were implemented. One uses 8 different green solvents for maximum yield of bioactive using green technology, and the other for the optimization of Microwave-assisted Extraction (MAE) parameters to enhance the bioactive components yield. Response Surface Methodology was employed to optimize the processing parameters including temperature, time, solid to solvent ratio, and solvent type. The optimized extraction conditions: treatment temperature of 50 °C for 5 min gave a significantly higher yield of total carotenoids (192.81 ± 0.32 mg carotenoids/100 g DW), total phenolic (78.12 ± 0.35 g GAE/100 g DW), and antioxidants by FRAP (5889.63 ± 0.47 mM TE/100 g DW), ABTS (1143.65 ± 0.81 mM TE/100 g DW), and DPPH (823.14 ± 0.54 mM TE/100 g DW) using a solvent combination of hexane and ethanol (1:3) with solid to solvent ratio of 1:40 (w/v). This green technology in combination with GRAS solvents promoted the best recovery of bioactive from carrot rejects. Moreover, the solid residue remained after the extraction of bioactive components exhibited higher carbon content (46.5%) and calorific value (16.32 MJ/kg), showcasing its potential to be used as an energy source.

## Introduction

Globally, over one-third of the total food produced is wasted along the food supply chain, which amounts to about 1.3 billion tons every year^1^. Fruits and vegetable waste are the most significant portion as this alone accounts for 40–50% of total solid waste generated^2^. In Canada, Ontario is the largest producer of fruits and vegetables accounting for 44% of the total production. Carrot is the second major field vegetable after tomato in Ontario with the highest farm gate value of CAD 129.3 million. Before reaching the consumers these carrots are subjected to stringent quality grading standards for size and shape, failing to match quality criteria leads to the generation of rejects/discards termed culled carrots or carrot rejects/waste^3^. As a result of this, carrot processing industries generate a large amount of waste of about one-quarter (25–30%) of the total harvest^4^. Besides economic loss, the disposal of these wastes into landfills also leads to serious environmental concerns due to the release of potent greenhouse gases including methane and carbon dioxide^5^. But the carrot rejects/wastes or by-products have the potential to be further processed into edible food or value-added products with considerable functional and nutraceutical benefits^6^.

Carrot processing by-products are unique due to their edibility and presence of high-value bioactive components such as dietary fibers, carotenoids, polyphenols, and antioxidants^7^. The valorization of these by-products can be achieved through the development of sustainable bioprocessing techniques utilized for the extraction of high-value bioactive components such as colorants, antioxidants, phytochemicals, and sequential processing into biofuels while adhering to the Zero-Waste circular economy principles^8^. Carrot is considered to be an excellent source of carotenoids^9^ and Carotenoids provide potential health-promoting functions such as antioxidant, antimutagenic, anti-inflammatory, immune-enhancement.

Most of the commercial and easily available carotenoids are synthesized by using chemical methods or extracted from plant sources using petrochemical solvents^10^. Carotenoids are soluble in non-polar solvents only, which limits the use of green food-grade polar solvents for their extraction^11^. On the other hand, conventional extraction with the use of petrochemical organic solvents has limitations such as prolonged extraction time, use of high temperature, hazardous volatile organic compounds emissions, low extraction yield, and higher residual waste^12^. To overcome these limitations, green extraction technologies can be used that are gaining interest due to their ability to provide high extraction yield, minimum degradation effect on thermo-sensitive bioactive components, lower processing temperature and time, and use of GRAS (Generally Recognized As Safe) solvents^13,14^.

Various green extraction technologies including Microwave-Assisted Extraction (MAE), Ultrasound-Assisted Extraction (UAE), Pulsed Electric Field (PEF), High Hydrostatic Pressure (HHP), and Supercritical Fluid Extraction (SFE) have been used for the extraction of bioactive components from fruit and vegetable by-products^12,15–17^. Out of all the aforementioned technologies, MAE is considered an emerging green extraction technology for thermolabile components^18^. The advantage of extraction using microwave power is that the cellular matrix is heated volumetrically without a significant thermal gradient that helps in the extraction of targeted components efficiently with minimal energy and solvent consumption^19^.

MAE has been successfully used for the extraction of bioactive components such as natural pigments, antioxidants, and phytochemicals from different agro-industry by-products^20,21^. But, limited research has been conducted on the application of MAE for green extraction of carotenoids using environment-friendly green solvents such as ethanol, ethyl acetate, ethyl lactate, etc.^22^. This study investigates the use of MAE in combination with green solvent for the extraction of high-value components from carrot processing rejects. The study also evaluates the structural morphology, elemental analysis, and calorific value of the extracted residual solid matrix for potential use as an energy source^8^.

## Materials and methods

### Chemicals and reagents

HPLC grade solvents such as Hexane, Acetone, ethanol, and food-grade ethyl acetate were obtained from Fisher Scientific (Ottawa, Ontario, Canada). Folin–Ciocalteu reagent, sodium bicarbonate (NaHCO_3_), Gallic acid, and methanol were procured from Fisher Scientific (Ottawa, Ontario, Canada). Standard β-carotene of 99% purity and 2,2-diphenyl-1-picrylhydrazyl (DPPH) were purchased from Sigma Aldrich (Sigma-Aldrich Canada Co., Oakville, Ontario, Canada). BHT (Butylated Hydroxy Toluene) was obtained from MP Biomedical, LLC (Illkirch, France).

### Raw material and sample preparation

Culled carrot waste including the whole jumbo carrots, rejected tops, and tips obtained during the processing of whole carrots (Varieties: Florida and Enterprise) were provided by Carrot growers and the processor (Smith Gardens, Ontario, Canada). The samples were cut into small pieces longitudinally, and stored at − 20 °C until further processing and analysis. The frozen carrots were freeze-dried for 48 h using a Freeze dryer (Harvest Right, USA). The freeze-dried samples were then ground and passed through a 300 µm sieve to obtain fine and homogenous particle size.

### Experimental design

Study 1 was conducted for the suitable selection of solvent for MAE of carotenoids from carrot rejects using single-factor experiments. In this study, three GRAS or food compatible solvents (Ethanol, Ethyl acetate, Acetone) and four solvent mixtures (Hexane/ethanol (1:1), Hexane/acetone (1:1), Hexane/ethyl acetate (1:1), Hexane/acetone/ethanol (2:1:1)) were selected for MAE of carotenoids. US Food and Drug Administration and US Health and Human Services have approved the three polar solvents (Ethanol, ethyl acetate, and acetone) as GRAS and environmentally safe^23^. These solvents are less toxic and have reduced risk to human health as compared to other organic solvents. According to the FDA, maximum exposure of 50 mg per day of these solvents is regarded as safe. On the other hand, the recommended safe exposure to hexane is about 2.9 mg per day^12^.

Study 2 studied the effect of different extraction process parameters including temperature, time, solvent mixture ratio, and solid to solvent ratio on the extraction yield of carotenoids from carrot rejects as well as bioactive components such as total phenols and antioxidants. These parameter values were identified by the preliminary study. The range of extraction parameters for the recovery of bioactive components was determined using one factor at a time while keeping others constant. In this study, the statistical significance of variables was chosen using a fractional factorial design with 4 variables. According to factorial design (2^4^ = 16) experiments with 4 center points were not feasible for this study due to more number of experimental sets so half fraction factorial (2^4–1^ = 8) with three center point replicates was considered (Table 1)^24^. The parameters (Temperature (40–70 °C), time = (5–15 min), Hexane/ethanol (25–100), and solid to solvent ratio (1:20–1:40 (w/v)) on the responses: Total carotenoid content, Antioxidant activity, and total phenols. Each experimental combination was replicated thrice, and analysis was performed using ANOVA.

**Table 1:** Factors and levels used in the MAE experimental design of bioactive components from carrot rejects.

Factors	Levels	
**Experiment 1: Preliminary experiments for solvent selection**		
Solvent type	8 Solvents (polar solvents and solvent mixture of polar and non-polar)	
	Ethanol, Ethyl acetate, Acetone, Hexane, Hexane/ethanol (1:1), Hexane/ethyl acetate (1:1), Hexane/acetone (1:1), Hexane/acetone/ethanol (2:1:1)	
**Experiment 2: Half Factorial design (2**^**4–1**^** factorial points + 3 center points)**		
Solvent type (Hexane:Ethanol)	25	100
Temperature (°C)	40	70
Time (min)	5	15
Solid:Solvent ratio (g/mL)	1:20	1:40
**Experiment 3: Rotatable CCD design for microwave digester process parameters optimization (FCCD with 3 factors and 5 levels + 5 center points)**		
Temperature (°C)	40	60
Time (min)	5	15
Solid:Solvent ratio (g/mL)	1:20	1:40

**Figure 1:**
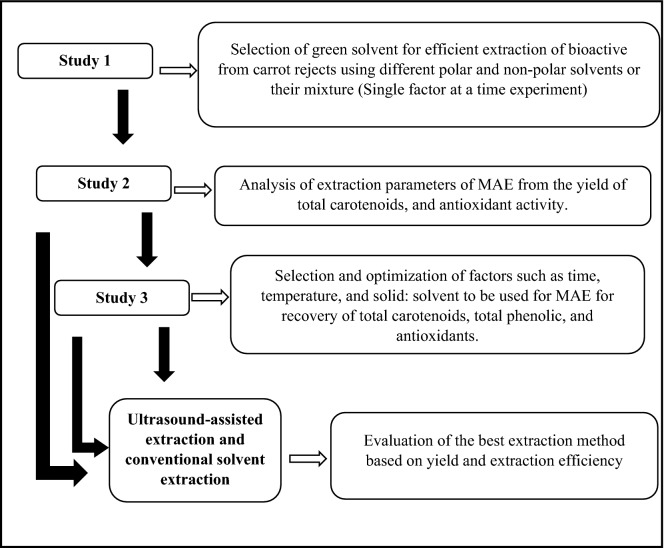
Experimental study plan of MAE of bioactive components from carrot rejects.

Study 3 studied the optimization of the significant factors obtained from factorial design in the previous study. In this study, an insignificant factor (solvent type) obtained in the fractional factorial study, was removed from the variables. To determine the optimum conditions for the maximum yield of carotenoids, total phenolic, and antioxidants, Rotatable CCD (Central Composite Design) was considered. Hence, in this study, three independent factors temperature (40–60 °C), time (5–15 min), and solid to the solvent ratio (20–40) were studied. Hence, the experiment consisted of 19 experimental runs containing a total of 8 factorial combinations, 6 axial, and 5 center point combinations (Fig. 1).

### Microwave-assisted extraction (MAE)

MAE of bioactive components such as carotenoids and antioxidants was performed in a closed multi-mode vessel Mini-Wave Microwave Digestion System (SCP Science, QC, Canada) working at 1000 W power and 2.45 GHz frequency working with focussed energy. Microwave digestion unit rack able to hold 6 quartz equidistant cylindrical vessels and six IR sensors were located on the sidewalls of digestion unit to monitor the temperature^25^. For microwave-assisted extraction, GRAS and food-grade solvents with high dielectric constants such as ethanol, acetone, and ethyl acetate are preferred. Hence, in this experiment, a solvent mixture of high and low dielectric constants such as polar and non-polar solvents were chosen to extract heat-labile components. Hexane, a non-polar solvent was used at a low proportion in combination with polar solvents of high dielectric power to prevent degradation and efficiently extract the non-polar components of carrot^10^.

For the experimental run, 1 g of powdered freeze-dried sample was taken in a quartz microwave digester tube mixed with 20 mL of the solvent. The samples were irradiated at a set temperature for a set time according to the experimental design. After MAE, the solvent mixture was filtered through Whatman filter paper and the solid residue was re-extracted with 20 mL fresh solvent under the same condition. The extracts from the two successive extractions were pooled together and centrifuged at 3500 rpm for 10 min to separate the supernatant. The separated supernatant was stored at 4 °C until further analysis.

### Conventional solvent extraction (CSE) and ultrasound-assisted extraction (UAE)

For CSE of carotenoids and antioxidants from carrot rejects, 1 g of freeze-dried powdered sample was blended with 40 mL corresponding solvent (hexane/ethanol 1:1) mixture and placed in a hot water bath at 50 °C for 1 h^26^. For the UAE, 1 g of freeze-dried carrot powder with 40 mL of solvent was treated with ultrasonic waves at a temperature of 50 °C for 5 min. Following the treatments, the same separation process was used as that for microwave-treated samples.

### Estimation of total carotenoids (TC) yield

The total carotenoid content of the extracted samples was measured by UV-spectrophotometer (Genesys 10S UV-spectrophotometer, Thermo-scientific, US)^27^. In the case of the polar and non-polar solvent mixture, the organic phase of the mixture was separated in a separatory funnel. Further, for TC analysis the supernatant of each sample was measured at the max wavelength (λ_max_) determined for carotenoids in each solvent or solvent mixture as blank. For the estimation of total carotenoid content in the extracted samples, the following equation was used, expressed in each extract:

1 $$ TCC = \frac{{{\text{A}}_{{\uplambda{\text{max}}}} *V*D*10^{4} }}{{A^{{1{\text{\% }}}} \;{\text{cm*W}}}} $$

where TCC is total carotenoid content expressed in µg/g DW of the sample, ${\text{A}}_{{\uplambda{\text{max}}}}$ is the absorbance of the extract at the maximum wavelength (λ_max_) determined for each solvent, V is the volume of the extract used, D is the dilution factor, 10^4^ is the conversion factor for concentration in µg/g, W is the weight (g) of the sample used for extraction, A^1%^_1 cm_ = extinction coefficient in different solvents.

### Antioxidant activity determination

#### FRAP assay

To determine the antioxidant activity of carrot rejects extract, a ferric ion reducing antioxidant power (FRAP) assay adapted from Hiranvarachat and Devahastin^28^ with some modifications was used. Trolox was used as standard and the calibration curve was plotted using various concentrations (0.02, 0.04, 0.06, 0.08, and 0.10 mg/mL) of the Trolox in ethanol against the corresponding absorbance at 593 nm with good linearity (y = 0.0094x + 0.1872, R^2^ = 0.99). For quantitative determination of antioxidant activity, 100 µL of carotenoid extract or standard solution was mixed with 3 mL of FRAP reagent and incubated at 37 °C for 30 min. After 30 min the absorbance of the samples was measured at 593 nm. The antioxidant content of the extract was expressed in terms of mM TE (Trolox Equivalent) per g dry weight of the sample.

#### ABTS assay

ABTS assay of the extracted carotenoid samples was performed according to Hiranvarachat and Devahastin with some modifications^28^. To determine the antioxidant activity, 100 µL of the sample was mixed with 2 mL of ABTS^+^ solution, kept in dark for 10 min, and then absorbance was measured at 734 nm using UV-spectrophotometer. The antioxidant activity of the extract samples was expressed as mM TE (Trolox equivalent).

#### DPPH assay

The in-vitro antioxidant activity of extracted carotenoids was assessed using the method suggested by^29,30^. In a typical experiment, an aliquot of 50 µL of the carotenoid extract was mixed with 1.5 mL of DPPH solution. This mixture was kept for incubation at room temperature in dark for 20 min. During this incubation period free radicals in DPPH get paired with antioxidants present in the carotenoid extract which further decrease the purple color of DPPH. This reduction in absorbance of samples due to the lightening of purple color gives the amount of antioxidant content of the extracted samples.

2 $$ Antioxidant\;\;activity\; \;(\% ) = 100 \times \;\left( {\frac{{Abs_{control}^{517nm} - Abs_{sample}^{517nm} }}{{Abs_{control}^{517nm} }}} \right) $$

where Abs _control_ is the absorbance of the control (without the sample) and Abs _sample_ is the absorbance of the sample.

### Total phenolic content (TPC)

The total phenolic content of the carrot extract was measured using Folin–Ciocalteu’s reagent with slight modifications^31^. 1 mL of the sample was mixed with 7.5 mL of distilled water and 0.5 mL of Folin–Ciocalteu’s reagent (diluted in 1:10 with water). The mixture was kept at room temperature for 5 min and 1 mL of 5% (w/v) sodium bicarbonate was added to the mixture. The mixture was kept at incubation for 90 min and absorbance was measured at 765 nm. Results for TPC were reported as g gallic acid equivalents (GAE)/100 g DW.

### HPLC (high-performance liquid chromatography)

For the identification of carotenoids, the extract obtained after extraction was subjected to RP-HPLC and was analyzed using the modified method^32^. The HPLC (Beckman Coultar Gold) system used for analysis consisted of 126 solvent pumps, 166 UV–vis detectors, 508 autosampler, and equipped with an RP-C18 Column (150 × 4.6 mm, 5 µm particle size) (Phenomenex, Torrance, US). Two mobile phases assayed for carotenoid identification were Mobile Phase A: Acetonitrile/Water/Glacial acetic acid (90/10/2), and Mobile Phase B: Methanol/Tetrahydrofuran (40/60). The gradient used was: 98% A and 2% B for 1–5 min, then increased to 80% B in the next 5 min, subsequently in the last 20 min B was increased to 100%. 20 µL of the sample was injected using a flow rate of 1 mL/min and carotenoids were detected at 450 nm. For the phenolic component analysis, the mobile phases used were Mobile Phase A: 5% Formic acid in water and Mobile Phase B: 20% Formic acid (5% v/v) + 80% Acetonitrile. The gradient elution used was: 0–5 min—100% A, at 20 min—95% A, at 50 min—85% A, at 75 min—75% A, at 90 min—65% A. In the end, at 95 min—100% B, and 105 min—100% A was used for flushing out all the components from the column. 20 µL of the sample was injected using a flow rate of 1 mL/min and different phenolics were detected at 280, and 320 nm^33^. The carotenoid and phenolic components in extract samples were identified by comparing the retention time and absorption spectra of the reference standards.

### Morphological analysis of microwave treated carrot powder

SEM (scanning electron microscopy) was used to study the effect of the microwave extraction process on the microstructure of the carrot reject residue. Micrographs of freeze-dried carrot powder before and after microwave treatment were obtained for morphological characterization and treatment effect on the cellular matrix. Four samples: freeze-dried carrot rejects powder (un-treated), MAE treated carrot powder, CSE treated carrot powder, and UAE treated carrot powder after the extraction of bioactive were taken and dried at 40 °C until the constant dry weight was achieved. For SEM analysis, a double-sided carbon tape placed on the surface of a clean metal stub was used over which all treated and non-treated dried powders were evenly distributed, and a thin layer of Au–Pd was sputter-coated to improve overall image quality. Dried carrot powder characteristics such as shape and surface features were analyzed at 10 kV accelerating voltage with a 500–2000 × magnification microscope (FEI Quanta, FEG 250 SEM)^12^.

### Elemental analysis and calorific value of biomass

After the extraction of carotenoids from carrot rejects under optimal conditions, the exhausted residues were recovered and dried at 110 °C. Following drying, elemental analysis was performed to determine the carbon, hydrogen, and nitrogen content of the samples. 2,5-Bis (5-tert-butyl-2-benzo-oxazole-2-yl) thiophene) (BBOT) was used as standard. All the analyses were performed in triplicates using Organic Elemental Analyzer CHN-O (Thermo Flash 2000, Thermo Fisher Scientific, USA). Oxygen percentage was calculated theoretically by taking sample moisture and ashes into account. Elemental analysis of untreated samples was also performed using the same conditions. Further, the calorific value of the exhausted biomass was determined using a bomb calorimeter (IKA-C200 bomb calorimeter) in which 0.5 g of the dried sample was treated at a reduced pressure of 30 bars and a sample was ignited to determine the calorific value^27^.

### Statistical analysis

All the experimental runs were replicated thrice, and results were presented as mean ± SD. ANOVA analysis of all the results with post hoc Tukey’s analysis was determined to evaluate the effect of factors on the total yield of bioactive components. Design-Expert software version 12 was used to construct and analyze the effects of the different factors in study 2 and study 3. Data obtained from CCD was statistically analyzed with p < 0.05 and p < 0.01 for significance.

## Results and discussion

### Analysis of study 1

This experimental study represented the results of a single-factor experiment carried out for preliminary optimization of solvent type for green recovery of bioactive components such as total carotenoids and antioxidants from freeze-dried carrot rejects using MAE. Table 2 presents the results of total carotenoids and antioxidants obtained using MAE treated at a temperature of 50 °C for 10 min, solid to solvent ratio of 1:20 in two successive extractions with different organic solvents or their mixtures. From the results, it can be estimated that a mixture of polar solvents with a pre-determined ratio (1:1) of a non-polar solvent (hexane) gave a comparatively improved yield of total carotenoids (160–173 mg/100 g DW) consisting of lipid-soluble (β-carotene and α-carotene) and lipophilic carotenoids than individual polar solvents (98–150 mg/100 g DW). All the solvents showed a statistically significant (p < 0.05) difference in total carotenoid yields from carrot rejects. The maximum yield of carotenoids (172.88 ± 3.07 mg/100 g DW) was obtained with hexane/ethanol mixture (1:1, v/v) followed by hexane/acetone/ethanol, 2:1:1 mixture (170.05 ± 1.66 mg/100 g DW). This can be explained by the fact that total carotenoids present in carrot tissues were majorly carotenes (β and α carotenes) along with xanthophyll (lutein), which can be completely extracted with polar and nonpolar solvent mixture from low moisture samples. Similar observations for the solvent suitability for carotenoid extraction have been reported by Lin and Chen^34^ where the maximum yield of carotenoids (lycopene) in tomato juice was obtained with an ethanol/hexane mixture (4:3 v/v).

**Table 2:** Total carotenoids and antioxidant activity of extract obtained from carrot rejects in study 1.

Solvent/solvent mixture	Maximum wavelength for carotenoid determination (nm)	Total Carotenoid content (mg/100 g DW)	ABTS assay (mM Trolox/100 g DW)	FRAP assay (mM Trolox/100 g DW)	DPPH (mM Trolox/100 g DW)
Ethanol	452	98.34 ± 4.04^a^	745 ± 17.38^d^	596.21 ± 97.44^a^	135.43 ± 21.73^f^
Ethyl acetate	454	154.95 ± 0.94^d^	639.44 ± 25.46^b^	648.89 ± 77.14^c^	153.21 ± 18.30^g^
Acetone	454	149.09 ± 4.04^b^	617.22 ± 41.94^a^	619.26 ± 19.75^b^	110.38 ± 12.20^e^
Hexane	450	151.59 ± 2.66^c^	991.11 ± 20.46^g^	2011.85 ± 98.76^g^	47.35 ± 28.31^a^
Hexane/acetone (1:1)	454	162.69 ± 3.52^e^	811.67 ± 15.18^f^	1794.57 ± 77.14^d^	93.41 ± 12.44^d^
Hexane/ethanol (1:1)	452	172.88 ± 3.07^h^	996 ± 26.38^h^	2234.48 ± 99.42^h^	58.66 ± 11.11^b^
Hexane/ethyl acetate (1:1)	454	168.52 ± 2.89^f^	650.55 ± 41.75^c^	1882.22 ± 29.63^e^	77.25 ± 25.69^c^
Hexane/acetone/ethanol (2:1:1)	450	170.05 ± 1.66^g^	789.44 ± 25.09^e^	1935.31 ± 52.26^f^	110.38 ± 22.91^e^

Values are presented as mean ± SD of three replicates.

Different letters in the same column indicate a statistically significant difference at (p < 0.05).

The antioxidant activity of the carotenoids extracted from carrot rejects was determined by FRAP, ABTS, and DPPH. Antioxidant activity obtained by FRAP with different solvents was in the range of 590–2240 mM TE/100 g DW. FRAP results of carotenoids extracted with different solvents revealed that Hexane/ethanol solvent extract gave the maximum yield of antioxidants (2234.48 ± 99.42 mM TE/100 g DW) followed by hexane, hexane/ethanol/acetone mixture. These results represented a direct correlation between total carotenoids and antioxidant activity. Hence, most of the antioxidant content of the extract is attributed to the presence of carotenoids majorly β-carotene present in carrots. Meanwhile polar solvent extracts also represented a higher concentration of antioxidants in the extract due to the presence of phenolic components in the peels of carrot^35^.

The results for antioxidant activity by ABTS were observed in the range of 615–1000 mM TE/100 g DW. Hexane/ethanol extract showed a maximum yield of antioxidants (996 ± 26.38 mM TE/100 g DW) followed by hexane, hexane/acetone, and hexane/acetone/ethanol mixtures. On the other hand, antioxidant activity measured by the DPPH method reported a very low amount of Trolox equivalent in the range of 47–150 mM TE/100 g DW. The low amount of Trolox equivalent for DPPH can be explained by the poor ability of carotenoids to scavenge DPPH free radicals^36^.

### Analysis of study 2

**Figure 2:**
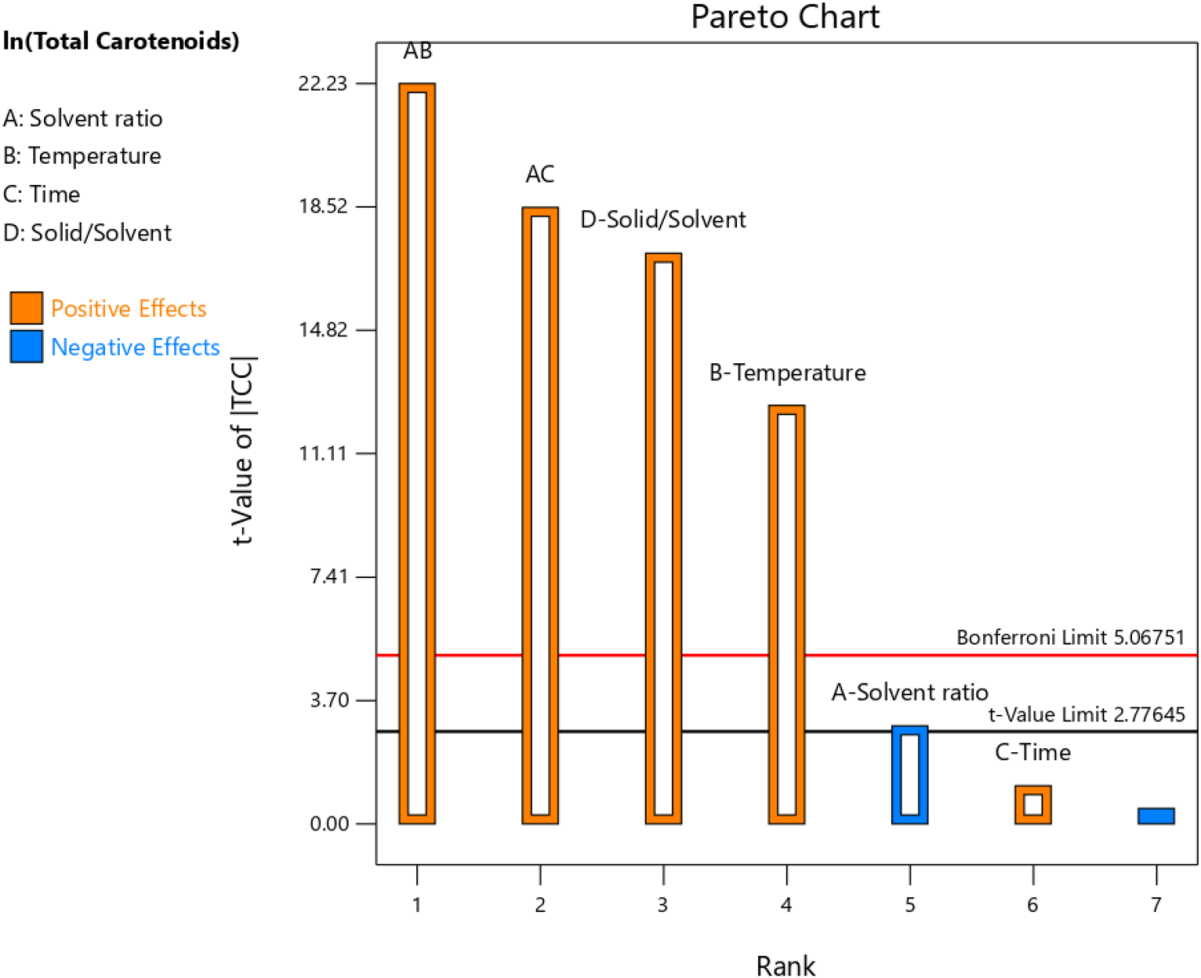
Pareto chart representation for the effect of different factors on Total carotenoids and antioxidant activity.

This study was done for the screening of MAE process parameters for the maximum yield of carotenoids and antioxidant activity by FRAP, ABTS, and DPPH extracted from carrot rejects using a factorial design (2^4–1^). The effect of each factor on the responses was evaluated at a 95% confidence interval using (p < 0.05). Total carotenoid content extracted from the carrot rejects using MAE in study 2 was found in the range of 78.55–221.76 mg/100 g DW was relatively higher than the amount reported in previous studies (58 ± 6 mg/100 g DW) (Table S1)^28^. But this higher yield of carotenoids (254.248 ± 3.89 mg/100 g DW) can be correlated with carotenoid content obtained from the freeze-dried powder of mixed cultivated carrots^37^. ANOVA analysis of this study revealed that temperature and solid/solvent ratio were found to be the most significant factors (p < 0.05) in obtaining a higher TCC yield (Table 3). However, solvent ratio and extraction time were found insignificant (p > 0.05). Interaction parameters such as solvent ratio with temperature and time were found significant (p < 0.05) (Table S2). In comparison to other factors, the solid to solvent ratio was the most significant factor for TCC (Fig. 2). A higher ratio of solid to solvent is preferred as it helps in easy permeation of bioactive components into the solvent, and consistent with the mass transfer phenomenon of higher concentration gradient with a more solvent ratio. Hence the results obtained in this study were in agreement with the reported results of Wong et al.^38^ in which maximum yield of total phenolic, flavonoids, and antioxidant activity was observed with a high solid to the solvent ratio (1:100 g/mL).

**Table 3:** ANOVA and regression analysis of Fractional factorial design responses of Study 2.

Source	Total carotenoid content	FRAP	ABTS	DPPH
	p-value	p-value	p-value	p-value
Model	0.0005*	< 0.0001*	0.0264*	< 0.0001*
A-Solvent ratio	0.0604	< 0.0001*	0.0630	< 0.0001*
B-Temperature	0.0011*	< 0.0001*	0.8881	< 0.0001*
C-Time	0.3362	0.0007*	0.0288*	0.0003*
D-solid/solvent	0.0004*	< 0.0001*	0.2240	< 0.0001*
AB	0.0002*	0.2345	0.0397*	0.0005*
AC	0.0003*	0.0071*	0.1890	0.0130*
AD	0.3126	0.0058*	0.0065*	0.0001*
Curvature	0.0014*	< 0.0001	0.1155	< 0.0001
Lack of fit	0.7328	0.1522	0.1434	0.234
R^2^	0.9277	0.9687	0.9659	0.9996
R^2^ adjusted	0.8931	0.9081	0.8977	0.9989

On the other hand, an increase in temperature negatively affects the carotenoid yield due to isomerization and thermal degradation of thermolabile components. The boiling temperature of the hexane/ethanol mixture was observed to be 59 °C, so the maximum temperature range should be kept relatively low (maximum 60 °C) for further optimization^39^. An increase in the ratio of hexane with ethanol represented a positive effect on the carotenoid yield. The solubility of carotenoids mainly depends on the ratio of non-polar solvents in the mixture due to their hydrophobic nature. Hence, the increase in hexane ratio in solvent mixture enhances the extraction efficiency of carotenoids with high mass transfer and effective diffusion rate^24^.

In the case of antioxidant activity determined by FRAP and ABTS, it was observed that antioxidant activity for FRAP was in the range of 853–4438.4 ± 28.98 mM TE/100 g DW and for ABTS 192–994.5 ± 17.98 mM TE/100 g DW is in correlation with the results reported for the intermittent MAE of carotenoids from pre-treated carrots^28^. ANOVA analysis for FRAP determination represented that all the factors caused a significant effect (p < 0.05) on the yield of antioxidants with a correlation coefficient of R^2^ = 0.9687. For the FRAP assay, the maximum antioxidant content was observed at the center point of all the factors which means that extraction at a high temperature for a prolonged time may ultimately affect the antioxidant activity of the extract. Whereas for ABTS antioxidant assay the extraction time was the only significant factor affecting the yield of antioxidants. Few interaction factors such as solvent ratio with temperature and solid to solvent ratio also showed a significant effect on the ABTS assay for antioxidants determination (p < 0.05). Solid to solvent ratio and time had a significant effect on the antioxidant activity by both ABTS and FRAP assays (p < 0.05) (Table 3).

DPPH radical scavenging activity of carrot rejects extract was observed in the range of 152–505.5 ± 23.43 mM TE/100 g DW. ANOVA analysis of the DPPH assay demonstrated that all the extraction parameters cause a significant effect (p < 0.05) on the antioxidant activity with a correlation coefficient of 0.919 which gave the significance of the model as well (Table 3). Hence it can be concluded that temperature, solid to solvent ratio, and extraction time were the significant factors that can be used with improved levels to optimize the yield of total carotenoids and antioxidant content in further study.

### Analysis of study 3

#### CCD model fitting

Study 3 was conducted to optimize the yield of total carotenoids, total phenolic, and antioxidants using a rotatable central composite design with three independent factors (temperature, time, and solid to solvent ratio) screened from the previous study. The analytical results of the CCD design experiment of carrot extract obtained with MAE parameters revealed a strong variability in total carotenoid content ranging between 96–301.28 mg carotenoids/100 g DW, total phenolic content (2.7–8.5 g GAE/100 g DW), and antioxidants estimation by FRAP (1740–8420 mM TE/100 g DW), ABTS (344–1363 mM TE/100 g DW), and DPPH (102–1035 mM TE/100 g DW).

#### Response surface analysis of total carotenoid content

The results of the CCRD analysis revealed that the carotenoid content extracted from carrots by MAE ranged between 96–301.28 mg carotenoids/100 g DW, clearly indicating the effect of MAE on different processing parameters on yield. The maximum yield of total carotenoids was obtained at extraction temperature of 50 °C, time of 10 min, and solid to solvent ratio of 1:46.8 (w/v), while the lowest yield was obtained at extraction temperature of 66.8 °C, time of 10 min, and solid to solvent ratio of 1:30 (w/v) (Table 4). In a study conducted by Hiranvarachat et al.^28^, the maximum carotenoids yield (219 mg/100 g d.b.) was obtained from carrot peels with the use of intermittent microwave radiations at 300 W microwave power and the solid-solvent ratio of 2:150 (w/v) with the solvent mixture (hexane:acetone:ethanol 2:1:1). The results obtained in study 3 revealed that with the selected MAE parameters, the carotenoid yield obtained was two to three-fold higher than the results reported in the literature with different extraction methods such as conventional and ultrasound^12,40^. ANOVA analysis of the response parameters for total carotenoids and their significance was determined by p-value and F-value. The transformed regression equation obtained from CCRD for the carotenoid yield is given as follows:

3 $$ {\text{ln }}\left( {{\text{TCC}}} \right) \, = { 5}.0{5 }{-} \, 0.{1}0{68}*{\text{A }} + 0.0{15}*{\text{B }} + \, 0.{2232}*{\text{C }}{-} \, 0.{1629}*{\text{AB}} $$

**Table 4:** Analytical results of the carrot extracts obtained under different conditions of MAE (R-CCD response analysis) (Study 3).

Run	Extraction conditions			Analytical determination responses				
	A: Temperature (°C)	B: Time (min)	C: Solid to solvent ratio	TCC (mg carotenoids/100 g DW)	TPC (g GAE/100 g DW)	FRAP (mM TE/100 g DW)	ABTS (mM TE/100 g DW)	DPPH (mM TE/100 g DW)
1	50	10	30	171.78 ± 4.41	4.11 ± 0.37	3598.14 ± 39.8	905.67 ± 2.45	247.65 ± 8.92
2	60	15	20	100.62 ± 3.78	3.59 ± 0.18	2746.51 ± 29.2	549.53 ± 2.56	509.18 ± 7.45
3	40	15	20	173.76 ± 4.81	3.71 ± 0.25	2278.99 ± 23.1	504.75 ± 2.34	273.87 ± 4.45
4	50	10	46.8179	301.99 ± 6.89	6.62 ± 0.08	4324.72 ± 12.1	1363.04 ± 3.67	1035.51 ± 3.68
5	60	15	40	185.03 ± 4.67	7.36 ± 0.45	5472.22 ± 15.8	967.79 ± 3.64	537.42 ± 2.89
6	50	10	13.1821	98.73 ± 1.38	2.71 ± 0.42	1392.47 ± 16.7	344.67 ± 4.53	387.21 ± 3.45
7	50	10	30	179.34 ± 2.69	4.28 ± 0.20	3487.13 ± 17.2	919.14 ± 3.89	276.09 ± 3.45
8	66.8179	10	30	96.68 ± 2.98	7.71 ± 0.28	2741.52 ± 12.3	739.91 ± 4.34	794.06 ± 2.98
9	50	10	30	111.07 ± 2.48	3.86 ± 0.14	3476.81 ± 11.8	926.91 ± 7.51	257.34 ± 3.41
10	40	5	20	104.93 ± 1.81	4.83 ± 0.37	3297.82 ± 23.4	583.04 ± 6.78	567.45 ± 4.56
11	60	5	40	194.44 ± 3.05	7.87 ± 0.72	5347.41 ± 21.1	928.82 ± 6.56	618.14 ± 3.67
12	50	10	30	118.39 ± 2.69	3.81 ± 0.73	4204.08 ± 22.1	939.86 ± 4.56	235.58 ± 3.43
13	60	5	20	177.39 ± 4.79	4.30 ± 0.08	2613.37 ± 21.2	429.88 ± 4.78	237.96 ± 4.56
14	50	10	30	166.25 ± 2.62	3.94 ± 0.42	3795.17 ± 21.5	932.13 ± 3.67	187.82 ± 5.21
15	50	18.409	30	171.74 ± 0.82	3.26 ± 0.27	4801.55 ± 22.3	913.44 ± 3.45	145.96 ± 3.67
16	50	1.59104	30	158.53 ± 3.05	3.84 ± 0.68	4322.14 ± 27.8	905.15 ± 4.56	102.65 ± 3.45
17	40	5	40	156.05 ± 3.18	8.59 ± 0.28	6735.58 ± 16.7	1101.81 ± 5.61	956.88 ± 3.15
18	40	15	40	187.19 ± 1.82	5.82 ± 0.56	4738.80 ± 12.3	854.97 ± 6.32	557.84 ± 2.78
19	33.1821	10	30	257.21 ± 4.20	7.29 ± 0.20	2846.97 ± 8.9	689.66 ± 5.61	632.63 ± 3.45

**Figure 3:**
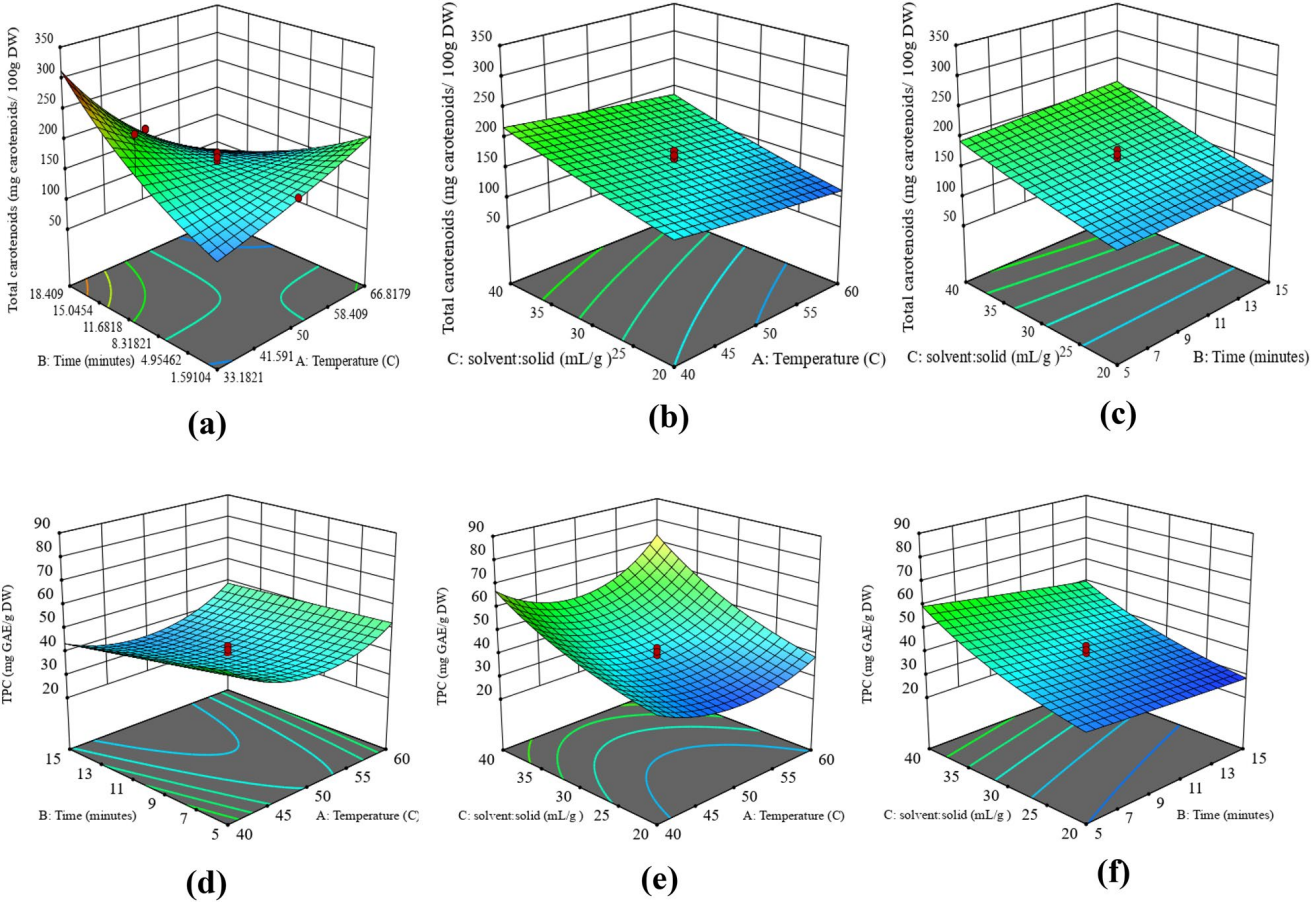
Response surface plots for representation of the combined effects of (**a**) A and B on TCC, (**b**) A and C on TCC (**c**) B and C on TCC, (**d**) A and B on TPC, (**e**) A and C on TPC (**f**) B and C on TPC (A: Temperature (°C), B: Time (min), C: Solid to solvent ratio (w/v)).

Results of the linear model indicated that the extraction factors (temperature and time) were not the significant factors (p > 0.05) for TCC. The coefficient of determination (R^2^ = 0.6973) was not in agreement with the adjusted (R^2^ = 0.5476), indicating the lower validation of model fit for biological materials^41^. However, the non-significant lack of fit (p > 0.05) for the model validates the influence of extraction parameters on the extraction yield. According to ANOVA results presented in Table S4(a), the solid to solvent ratio is the only significant factor that had a positive linear effect on total carotenoids yield. On the other hand, temperature and time were non-significant linear factors (p > 0.05), while the interaction among them was significant (p < 0.05). Figure 3a represented the interaction effect of time and temperature on TCC. Highest TCC was obtained at a high-temperature short time and a low-temperature long time of microwave extraction. Hence, from the results, it can be concluded that TCC yield increased with an increase in extraction time at moderate temperatures and reduced with an increase in the time at higher temperatures. A study conducted by^42^ also revealed that carotenoids recovery from tomato peels decreased after treating the material at a higher temperature for a prolonged time. However solid to solvent ratio significantly affected the TCC yield. With the increase in solid to solvent ratio from 1:10 to 1:40 (w/v), the carotenoid yield was observed to be increased due to the concentration gradient (Fig. 3b,c). This observation can be supported by the understanding that with an increase in the solvent ratio, the mass transfer and solubility of each component in the solvent increases allowing the release of components from the solid matrix into the solvent until it reaches equilibrium. The results obtained in this study on the solid to solvent ratio were related to the results obtained for the extraction of carotenoids from cantaloupe waste^12^. Hence, the optimized conditions for maximum yield of TCC were temperature of 40 °C, time 15 min, and solid/solvent 1:40 (w/v).

#### Response surface analysis of total phenolic content

Total polyphenol content in carrot extract obtained using MAE varied from 27–86 mg GAE/g DW. The maximum extraction yield for TPC (85.98 mg GAE/g DW) was obtained at a temperature—40 °C, time of 5 min, and a solid to solvent ratio of 1:40 (w/v). A two to three-fold increase in extraction yield of total phenolic components from carrots was observed in this study in comparison to yields reported in the literature^43,44^.

The regression equation defining the effect of statistically significant variables on the yield of TPC is represented as follows:

4 $$ {\text{ln}}\left( {{\text{TPC}}} \right) \, = { 3}.{667 } + \, 0.00{66}*{\text{ A }} - 0.0{859}*{\text{ B }} + \, 0.{2821}*{\text{ C }} + \, 0.{2497}*{\text{ A}}^{{2}} + \, 0.0{471}*{\text{ C}}^{{2}} $$

Results of the quadratic reduced model indicated that the relationship between the extraction factors and TPC had a good correlation coefficient (R^2^ = 0.9487) and the non-significant lack of fit (p > 0.05) validates the model. Among the different extraction parameters, TPC yield was significantly affected by time and solid to solvent ratio (linear effect) as well as the positive quadratic effect of temperature. None of the interactive effects of any parameters significantly affected the yield of total phenolic components (Table S4(b)).

Figure 3d–f represents the interaction between solid to solvent ratio, temperature, and time. The extraction yield of phenolic components increased with an increase in solid to solvent ratio from 20 to 40 mL per gram of solids as the concentration gradient increased with an increase in solvent volume and facilitate the diffusion of phenolic components into the solvent with effective mass transfer^45^. Further, extraction time also affected the yield of TPC as the maximum yield was observed until the exposure time of 5–7 min, thereafter additional time exposure caused a decrease in the yield of polyphenols at higher temperatures. ANOVA analysis proved that recovery of total phenolics was mainly dependent on the linear effect of time and solid to solvent ratio (p < 0.01) and quadratic effect of temperature that resulted in a curvilinear increase in total phenolics for all the temperature and time conditions (Table S3) (Fig. 3d,f). The microwave treatment of high temperature enhanced the yield of phenolic compounds, as high temperature reduces the viscosity of solvent and helped in easy release and dissolution of components into the solvent. However, exposure to high temperatures for a prolonged time leads to the degradation of phenolic compounds and results in a decrease in the yield of total phenolic^46^.

Hence, from the presented results, the optimized conditions for the maximum yield of total phenolic content (76.92 mg GAE/g DW) were microwave temperature of 50 °C, time 5 min, and solid to solvent ratio (1:40).

#### Response surface analysis of antioxidant activity (FRAP, ABTS, and DPPH)

The response analysis study for the antioxidant activity was measured by FRAP, ABTS, and DPPH assays. Total antioxidant activity measured by FRAP, ABTS, and DPPH in carrot extract obtained using MAE varied from (1392–6735 mM TE/100 g DW), (344–1363 mM TE/100 g DW), and (102–1036 mM TE/100 g DW) respectively. The variation in the yield of antioxidants in all the analyses revealed that extraction parameters significantly affected the yield.

The quadratic polynomial equations with significant terms for antioxidant activity of carrot extract obtained with MAE are given below:

5 $$ {\text{ln }}\left( {{\text{FRAP}}} \right) = { 8}.{1587 } - 0.0{1438}0{3 }*{\text{ A }} - 0.0{345235 }*{\text{ B }} + \, 0.{348356 }*{\text{ C }} + \, 0.0{99239 }*{\text{ AB }} + \, 0.{127218 }*{\text{ B}}^{{2}} - 0.0{915}0{14 }*{\text{ C}}^{{2}} $$

6 $$ {\text{ln }}\left( {{\text{ABTS}}} \right) = { 6}.{81232 } - 0.0{1}0{86}0{2 }*{\text{ A }} - 0.00{7}0{183 }*{\text{ B }} + \, 0.{352357 }*{\text{ C }} + \, 0.0{855618 }*{\text{ AB }} - 0.{1}0{3597 }*{\text{ A}}^{{2}} - \, 0.{118211 }*{\text{ C}}^{{2}} $$

7 $$ {\text{ln }}\left( {{\text{DPPH}}} \right) = { 5}.{46737 } - 0.0{249641 }*{\text{ A }} - 0.00{4}0{5198 }*{\text{ B }} + \, 0.{285343 }*{\text{ C }} + \, 0.{2361}0{9 }*{\text{ AB }} + \, 0.{432696 }*{\text{ A}}^{{2}} - 0.{188213 }*{\text{ B}}^{{2}} + \, 0.{392845 }*{\text{ C}}^{{2}} $$

Table 5 represented the regression values for antioxidant activity and the relationship between different extraction parameters and stated that all the parameters represented good correlation coefficient and non-significant lack of fit indicated that the model fits the experimental data adequately.

**Table 5:** ANOVA and regression analysis of CCD responses of Study 3.

Source	Total carotenoids content	TPC	FRAP	ABTS	DPPH
**Model**	0.0135*	< 0.0001*	< 0.0001*	< 0.0001*	< 0.0001*
A-Temperature	0.1193	0.7951	0.7228	0.6371	0.7146*
B-Time	0.8196	0.0045*	0.4004	0.7598	0.9525*
C-Solvent:Solid	0.0038*	< 0.0001*	< 0.0001*	< 0.0001*	0.0013*
AB	0.0733	–	0.0792	0.0129*	0.0200*
BC	–	–	–	–	–
AC	–	–	–	–	–
A^2^	–	< 0.0001*	–	0.0005*	< 0.0001*
B^2^	–	–	0.0070*		0.0164*
C^2^	–	0.0796	0.0377*	0.0002*	0.0001*
Lack of fit	0.4846	0.0676	0.0782	0.0813	0.1051*
R^2^	0.6973	0.9487	0.8931	0.9615	0.9117
R^2^ adjusted	0.5476	0.9289	0.8396	0.9423	0.8555

**Figure 4:**
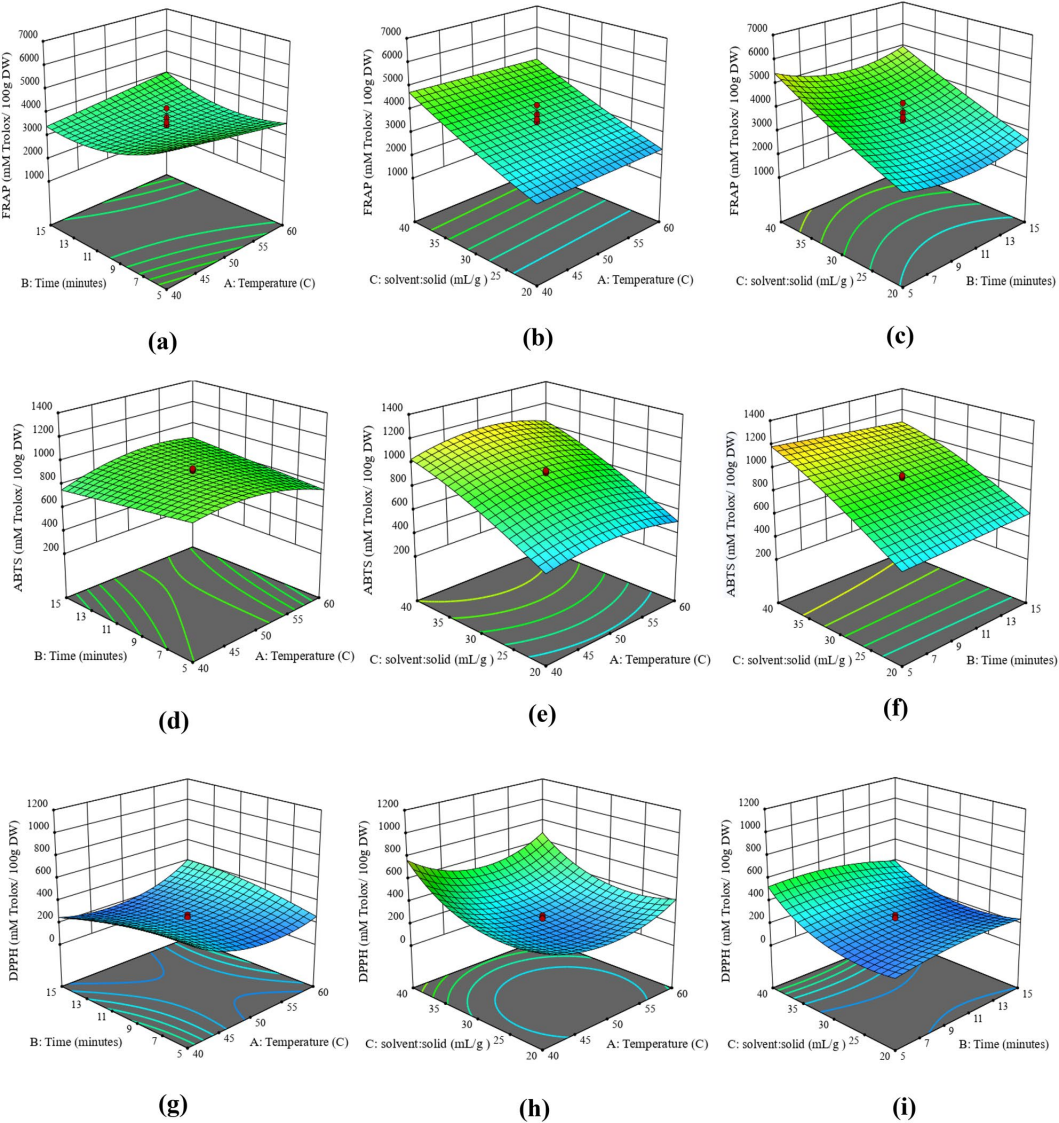
Response surface plots for representation of the combined effects of (**a**) A and B on FRAP, (**b**) A and C on FRAP (**c**) B and C on FRAP, (**d**) A and B on ABTS, (**e**) A and C on ABTS (**f**) B and C on ABTS, (**g**) A and B on DPPH, (**h**) A and C on DPPH (**i**) B and C on DPPH (A: Temperature (°C), B: Time (minutes), C: Solid to solvent ratio (w/v)).

The maximum antioxidant content by FRAP (6735.58 ± 16.37 mM TE/100 g DW) was obtained at a temperature of 40 °C, time 5 min, and solid to solvent ratio of 1:40 (w/v). These results for the antioxidant activity were correlated with the TPC. This can be explained by the fact that carotenoids and phenolic components collectively gave the total antioxidant activity of the substance, FRAP reacts at lower pH of 3.6 for short time, and oxidizes molecules to give antioxidants. Thus in carrot extract, phenolic compounds were reduced to antioxidants more easily than the carotenoids under acidic conditions, which gave total antioxidant activity^28^. The linear effect of solid to solvent ratio and interactive effect of temperature and time significantly affected the total antioxidant activity by FRAP (Table S4(c)). Figure 4a–c represented the interaction of different parameters for total antioxidant activity by FRAP. Figure 4a showed the interaction between time and temperature, at a lower temperature, an increase in extraction time led to a gradual increase in antioxidant content measured by FRAP, and at a lower temperature prolonged extraction time gave a higher FRAP value. This observation can be supported by the fact that a higher temperature and short time is more effective for the extraction of antioxidants^47^.

Further, a solid to solvent ratio of 1:40 is preferred for the extraction of antioxidants for the entire range of temperature and time. It was observed that a higher solid to solvent ratio enhanced the extraction of components from the solid and increase the dissolution at different temperatures and times (Fig. 4b,c). Hence from the analysis of the regression model, the optimal extraction conditions for maximum yield for FRAP values were microwave temperature of 50 °C, time 5 min, and solid to a solvent ratio (1:40).

The maximum antioxidant content by ABTS (1363 ± 3.67 mM TE/100 g DW) was obtained at a temperature of 50 °C, time of 10 min, and solid to solvent ratio of 1:46.8 (w/v) while the lowest yield (344.67 ± 4.53 mM TE/100 g DW) at 50 °C temperature, time 10 min, and solid to solvent ratio of 1:13.2 (w/v). The results for ABTS were correlated with the total carotenoid content. This can be explained by the fact that ABTS determination is based on the SET mechanism that works at pH around 7.4 that oxidizes the molecules to yield antioxidants. ABTS assay also measured the complex and individual components in the mixture and gave total antioxidant content^39^. Figure 4d–f showed the interaction of time, temperature, and solid to solvent ratio on ABTS value. In general, the interaction results for ABTS were similar to that of FRAP. At fixed conditions of temperature and time, an increase in solid to solvent ratio (1:40) gave the maximum ABTS value (Fig. 4e,f). Further interaction of time and temperature significantly affected the ABTS value (Table S4(d)). A lower range of temperature with a lower extraction time was more effective in the extraction of antioxidants from carrot rejects^48^. Hence the extraction conditions of microwave temperature of 40 °C, time of 5 min, and solid to solvent ratio (1:40) were the optimized results for the maximum ABTS value.

The maximum antioxidant content by DPPH (1035.5 ± 3.65 mM TE/100 g DW) was obtained at a temperature of 50 °C, time of 10 min, and solid to solvent ratio of 1:46.8 (w/v). ANOVA results of DPPH proved that temperature, time, and solid to solvent ratio all significantly affected the antioxidant content of DPPH (Table S3). The interaction effect of temperature and time also affected the DPPH value (Table S4(e)). Further, DPPH gave less antioxidant content as compared to other assays due to the insoluble nature of the non-polar components in DPPH solution. Moreover, the interaction factor among different factors defined the same relation as in FRAP and ABTS (Fig. 4 (g-i)). Low extraction temperature with low extraction time resulted in higher neutralization of the DPPH radicals into the solvent and enhanced the antioxidant activity measured by the DPPH assay ^49^. Hence the extraction conditions of microwave temperature of 40 °C, time of 5 min, and solid to solvent ratio (1:40) were the optimized results for the maximum DPPH value.

### Optimization of extraction factors and validation of the model

In study 3, the conditions for MAE of bioactive components from carrot rejects were optimized to maximize the yield of Total carotenoids, Total polyphenols, and Total antioxidants by FRAP, ABTS, and DPPH. For the optimization at a maximum yield of all the parameters Derringer’s desirability prediction method was used and the results were represented in Table 6. The optimized factors for the maximum yield of bioactive components were Temperature of 50 °C, time of 5 min, and Solid to the solvent ratio (1:40 w/v). For these optimized parameters the predicted values for TCC, TPC, and antioxidant activity by FRAP, ABTS, and DPPH were determined by Design-Expert software and presented in Table 6. Further, these predicted values were compared with the experimentally determined values obtained at the optimized condition with triplicate runs of the sample. These experimental results lie in the range of 95% CI of the predicted values which defined a good correlation of results and proved the suitability of the model to experimental conditions as well. Hence, the developed models for the optimization of MAE parameters for maximizing the yield of bioactive components validated the experimental results and were defined as a significant and adequate model.

**Table 6:** Representation of predicted and experimental values of the responses at optimized MAE conditions.

Analysis	Predicted value	95% PI low	95% PI high	Experimental observed value
Total carotenoids	186.79	135.422	257.642	192.81 ± 0.32
TPC	75.9092	68.2151	84.4712	78.12 ± 0.35
FRAP	6012.65	4902.28	7374.52	5889.63 ± 0.47
ABTS	1152.57	1026.64	1293.94	1143.65 ± 0.81
DPPH	800.612	563.886	1136.72	823.14 ± 0.54

### Comparison of MAE, UAE, and CSE

While comparing the extraction efficiency of MAE, UAE, and CSE at optimum conditions (temperature 50 °C, time 5 min, and solid to the solvent ratio (1:40), MAE recovered maximum yield of bioactive components (78% dry basis). It also gave 50–55% more recovery of the components when compared with UAE and CSE (Table 7).

**Table 7:** Comparison of the extraction yield of TC, TPC, and antioxidants activity (FRAP, ABTS, DPPH) of carrot rejects extracts obtained with MAE, UAE, and CSE.

Analysis	Experimental conditions	TC (mg carotenoids/100 g DW)	TPC (g GAE/100 g DW)	FRAP (mM TE/100 g DW)	ABTS (mM TE/100 g DW)	DPPH (mM TE/100 g DW)
MAE	50 °C, 5 min, and solid to solvent (1:40)	192.81 ± 0.32	78.12 ± 0.35	5889.63 ± 0.47	1143.65 ± 0.81	823.14 ± 0.54
UAE	50 °C, 5 min, and solid to solvent (1:40)	108.31 ± 0.45	58.72 ± 0.31	4771 ± 0.34	780.34 ± 0.56	667.05 ± 0.35
CSE	50 °C, 1 h, and solid to solvent (1:40)	97 ± 0.91	68.71 ± 0.87	3998 ± 0.45	670 ± 0.34	570.23 ± 0.67

### Characterization of bioactive components by HPLC

**Figure 5:**
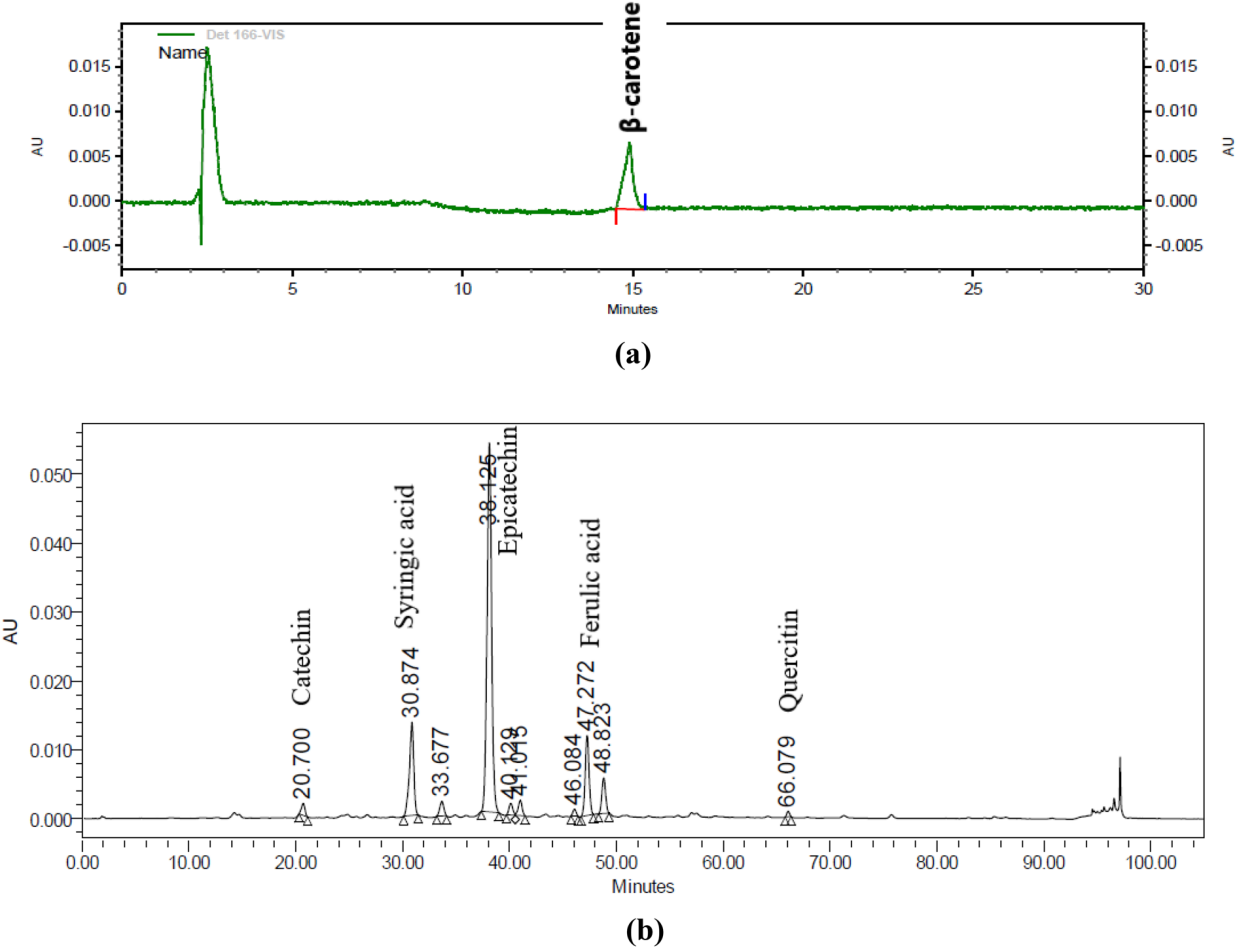
Characterization of (**a**) carotenoids and (**b**) phenolics using RP-HPLC.

HPLC analysis of the extract was carried out to identify the major carotenoids and phenolic components. The identification of the components was done by comparing the retention time and spectral data of the chromatograms with the standard carotenoid and phenolic components chromatograms. The peak obtained at 14.8 min with vis-detector at 450 nm wavelength represented β-carotene (Fig. 5a). A similar observation was also reported by Li et al. in tomatoes for lutein, lycopene, and carotenoids^32^. The peaks obtained by the UV-detector at 280 nm represented phenolic compounds—Rt: 20.7—Catechin, 30.8—Syringic acid, 38.12—Epicatechin, 47.27—Ferulic acid, 66.07—Quercitin (Fig. 5b). All these identified compounds are highly correlated with the higher antioxidant activity of the carrot extract. Similar results for the phenolic components in the cherry extract using a UV detector were reported by^50^. Hence these identified components suggested that the extraction using MAE with green solvents was successful for the extraction of components of interest.

### Assessment of structural morphological changes by SEM

**Figure 6:**
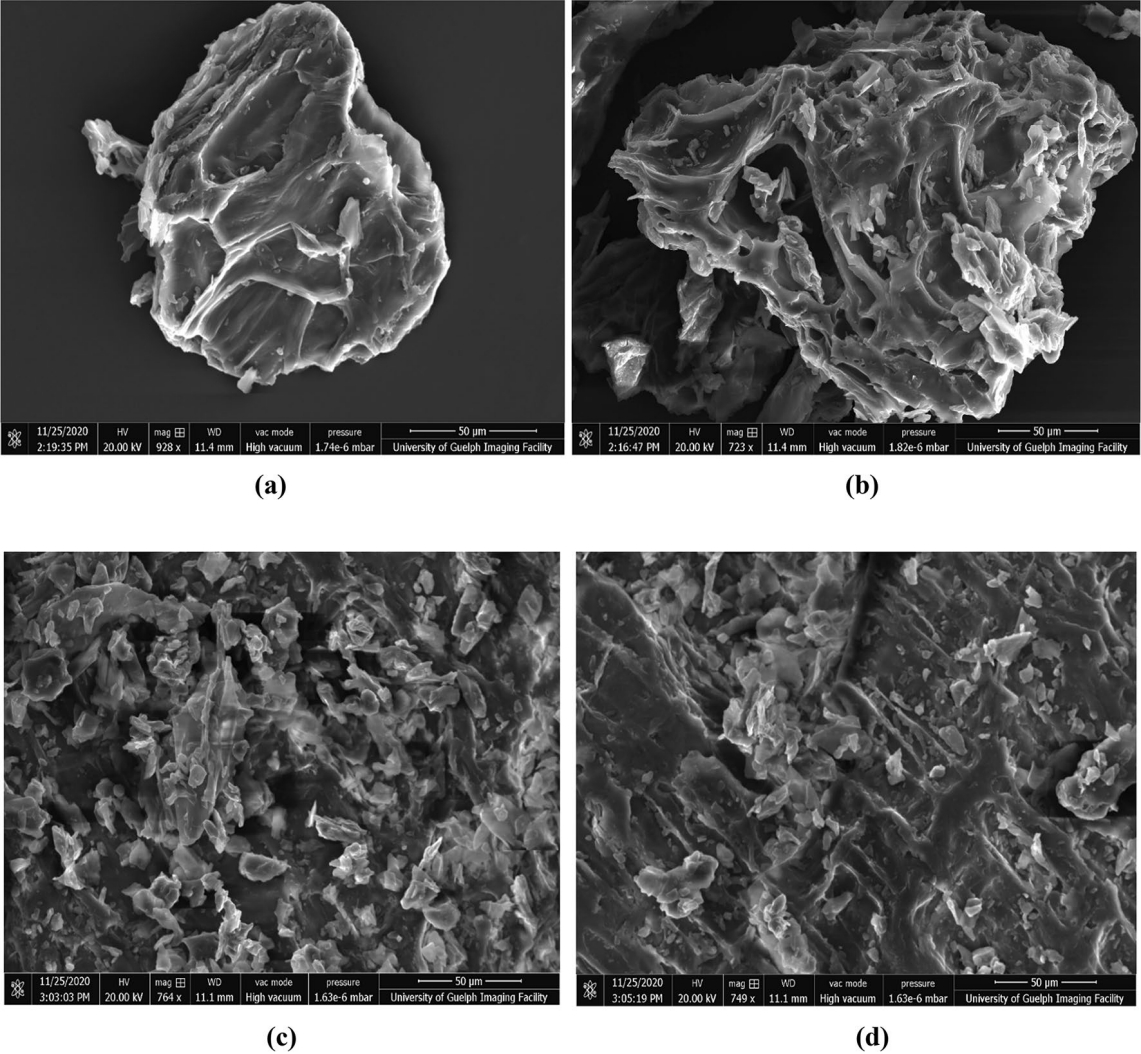
SEM micrographs of (**a**) Carrot powder untreated, (**b**) Maceration, (**c**) MAE, (**d**) UAE.

The micrographs of non-extracted carrot powder and powder extracted by MAE, UAE, and CSE were presented in Fig. 6. The micrographs of untreated powder and powder treated with different extraction techniques were taken at 500–100 × magnification and their structures were compared. Unlike the untreated carrot rejects powder that is completely imperforate and intact, the images of the extracted carrot powder presented a ruptured cell structure, which was abruptly damaged due to different treatments (MAE, UAE, and CSE) (Fig. 6a–d). Among all the micrographs, the carrot powder treated with MAE represented a fully disrupted structure that affected the integrity of the cellular matrix and facilitated better extraction of carotenoids and antioxidants (Fig. 6c). MAE treatment for 10 min encompasses erosion, fragmentation, capillary effect, and detexturation mechanism on the microscopic level for better extraction of components. The first erosion mechanism started with the addition of solvent, further with the microwave irradiation treatment fragmentation and detexturation occurred that helped in extraction of bioactives in solvent^51^. The pores of carrot powder treated with UAE were small and presented a wider surface area as compared to MAE treated powder (Fig. 6d). On the other hand, the micrograph of CSE treated carrot powder demonstrated very little damage to the tissue structure which in turn resulted in a lower extraction yield of bioactive components (Fig. 6b). From the results, it can be concluded that MAE generated more pores and fractures that helped in an easy diffusion of the solvent into the cellular matrix and release of solutes (bioactive components) into the extraction solvent (Fig. 6c). The results obtained in this study were completely related to the morphology of olive leaves treated with different extraction techniques i.e. Maceration, UAE, and MAE^25^.

### Elemental analysis

The solid residue remained after the extraction of bioactive components was analyzed for elemental composition. The CHN-O elemental analysis of the untreated carrot powder and microwave treated powder was conducted to determine the change in cellular matrix composition and the results obtained are presented in Table S4. The oxygen content of the residues was calculated theoretically from the elemental composition (CHN), moisture, and ash content of the biomass.

The solid residues obtained after the extraction of bioactive components by MAE under the optimized conditions contained 46.5 ± 0.05% C, 6.46 ± 0.41% H, 0.97 ± 0.13% N, and 46.07 ± 0.03% O, while the untreated carrot powder contained 40.2 ± 0.07% C, 6.26 ± 0.039% H, 0.94 ± 0.03 N, and 52.60 ± 0.02% O. It was observed that carbon and oxygen were the major elements of the solid residues followed by hydrogen and nitrogen. The results also suggested that there was an increase in carbon and hydrogen percentage of MAE treated carrot powder as compared to the untreated carrot powder leading to an increase in the calorific value from 15.78 to 16.32 MJ/kg. Similar results were reported for microwave-treated apple skins after the extraction of polyphenols^8^. The authors reported that the solid apple skin residue left after the extraction had a higher carbon content of 52.7% and calorific value of 24.6 MJ/kg, suggesting that the spent residue could be used as an energy source, supporting the notion of Zero Waste economy.

## Conclusion

The present study was conducted to utilize the carrot rejects and by-products for the extraction of high-value components such as carotenoids, polyphenols, and antioxidants. For the extraction of these bioactive components, MAE in combination with green solvents was employed. RSM design was used to determine the best optimal conditions for the high recovery of these components. The optimized process conditions of temperature 50 °C, time 5 min, and solid to solvent ratio of 1:40 (w/v) provided the excelling extraction yield values of TCC 192.81 ± 0.32 mg carotenoids/100 g DW, TPC 78.12 ± 0.35 g GAE/100 g DW, and antioxidants by FRAP 5889.63 ± 0.47 mM TE/100 g DW, ABTS 1143.65 ± 0.81 mM TE/100 g DW, and DPPH 823.14 ± 0.54 mM TE/100 g DW. The study also revealed that the spent extract residue can be used potentially as an energy source due to its high calorific value of 16 MJ/kg providing an opportunity to establish a sustainable zero-waste handling system for agricultural by-products.

## Data Availability

All the datasets used or generated during this study are included in this manuscript and supplementary data files.

## Supplementary Tables

Supplementary Tables.
