# Supplementary material for: Green extraction of bioactive components from carrot industry waste and evaluation of spent residue as an energy source
Source: Sci Rep. 2022 Oct 5;12:16607. doi: 10.1038/s41598-022-20971-5 (PMC9534898; doi:10.1038/s41598-022-20971-5)
Supplement: Supplementary file 1 — Supplementary Tables. [file 41598_2022_20971_MOESM1_ESM.docx]

**Supplementary Tables**

**Table S1: Factorial design used for estimation of total carotenoids and antioxidant activity by FRAP, ABTS, and DPPH**

| **Run** | **A:**  **Solvent ratio** | **B: Temperature** | **C: Time** | **D: Solid/ Solvent** | **Total Carotenoid**  **Content**  **(mg carotenoids/100g DW)** | **FRAP**  **(mM TE/100g DW)** | **ABTS**  **(mM TE/100g DW)** | **DPPH (mM TE/100g DW)** |
| --- | --- | --- | --- | --- | --- | --- | --- | --- |
| 1 | 25 | 40 | 15 | 40 | 131.48 | 3225.82 | 355.76 | 222.7 |
| 2 | 25 | 40 | 5 | 20 | 130.92 | 1981.16 | 994.75 | 165.09 |
| 3 | 25 | 70 | 15 | 20 | 78.55 | 2840.42 | 428.83 | 205.89 |
| 4 | 100 | 70 | 5 | 20 | 111.65 | 1094.39 | 345.34 | 333.98 |
| 5 | 100 | 40 | 5 | 40 | 79.27 | 1261.79 | 541.84 | 417.45 |
| 6 | 62.5 | 55 | 10 | 30 | 138.58 | 4438.4 | 368.58 | 196.80 |
| 7 | 62.5 | 55 | 10 | 30 | 147.54 | 4425.52 | 388.04 | 198.10 |
| 8 | 62.5 | 55 | 10 | 30 | 143.91 | 4403.08 | 328.27 | 198.70 |
| 9 | 100 | 40 | 15 | 20 | 83.87 | 853.12 | 192.14 | 152.97 |
| 10 | 25 | 70 | 5 | 40 | 152.06 | 3780.32 | 435.12 | 332.61 |
| 11 | 100 | 70 | 15 | 40 | 221.76 | 2320 | 606.33 | 505.53 |

**Table S2: ANOVA and regression analysis of Fractional factorial design responses of Study 2**

**(a) Response 1: Total Carotenoids content**

| **Source** | **Total carotenoid content** | | | | | |
| --- | --- | --- | --- | --- | --- | --- |
|  | **Sum of Squares** | **df** | **Mean Square** | **Coefficient estimate** | **F-value** | **p-value** |
| Model | 0.9268 | 6 | 0.1545 |  | 216.24 | 0.0005* |
| A-Solvent ratio | 0.0062 | 1 | 0.0062 | -0.0278 | 8.65 | 0.0604 |
| B-Temperature | 0.1127 | 1 | 0.1127 | -0.1187 | 157.72 | 0.0011* |
| C-Time | 0.0009 | 1 | 0.0009 | 0.0108 | 1.31 | 0.3362 |
| D-Solid/Solvent | 0.2095 | 1 | 0.2095 | 0.1618 | 293.28 | 0.0004* |
| AB | 0.3529 | 1 | 0.3529 | 0.2100 | 494.03 | 0.0002* |
| AC | 0.2446 | 1 | 0.2446 | 0.1749 | 342.43 | 0.0003* |
| Curvature | 0.0938 | 1 | 0.0938 | 0.0181 | 131.31 | 0.0014* |
| Residual | 0.0021 | 3 | 0.0007 |  |  |  |
| Lack of Fit | 0.0002 | 1 | 0.0002 |  | 0.1538 | 0.7328 |
| Pure Error | 0.0020 | 2 | 0.0010 |  |  |  |
| R^2^ | 0.9977 |  |  |  |  |  |
| R^2^ adjusted | 0.9931 |  |  |  |  |  |

**(b) Response 2: FRAP**

| **Source** | **Antioxidant activity determination (FRAP )** | | | | | | |
| --- | --- | --- | --- | --- | --- | --- | --- |
|  | **Sum of Squares** | **df** | **Mean Square** | **Coefficient estimate** | | **F-value** | **p-value** |
| Model | 7.930E+06 | 6 | 1.322E+06 |  | 1745.53 | | < 0.0001* |
| A-Solvent ratio | 4.959E+06 | 1 | 4.959E+06 | -787.30 | 6549.18 | | < 0.0001* |
| B-Temperature | 9.202E+05 | 1 | 9.202E+05 | 339.16 | 1215.35 | | < 0.0001* |
| C-Time | 1.573E+05 | 1 | 1.573E+05 | 140.21 | 207.72 | | 0.0007* |
| D-Solid/Solvent | 1.823E+06 | 1 | 1.823E+06 | 477.36 | 2407.61 | | < 0.0001* |
| AC | 32791.04 | 1 | 32791.04 | 64.02 | 43.31 | | 0.0071* |
| AD | 37851.01 | 1 | 37851.01 | -68.79 | 49.99 | | 0.0058* |
| Curvature | 1.107E+07 | 1 | 1.107E+07 | 2252.71 | 14623.16 | | < 0.0001 |
| Residual | 2271.47 | 3 | 757.16 |  |  | |  |
| Lack of Fit | 1632.49 | 1 | 1632.49 |  | 5.11 | | 0.1522 |
| Pure Error | 638.98 | 2 | 319.49 |  |  | |  |
| R^2^ | 0.9987 |  |  |  |  | |  |
| R^2^ adjusted | 0.9981 |  |  |  |  | |  |

**(c) Response 3: ABTS**

| **Source** | **Antioxidant activity determination (ABTS )** | | | | | |
| --- | --- | --- | --- | --- | --- | --- |
|  | **Sum of Squares** | **df** | **Mean Square** | **Coefficient estimate** | **F-value** | **p-value** |
| Model | 1.56 | 6 | 0.2604 | 6.09 | 14.16 | 0.0264* |
| A-Solvent ratio | 0.1535 | 1 | 0.1535 | -0.1385 | 8.35 | 0.0630 |
| B-Temperature | 0.0004 | 1 | 0.0004 | 0.0073 | 0.0234 | 0.8881 |
| C-Time | 0.2875 | 1 | 0.2875 | -0.1896 | 15.64 | 0.0288* |
| D-Solid/ Solvent | 0.0429 | 1 | 0.0429 | 0.0732 | 2.34 | 0.2240 |
| AB | 0.2241 | 1 | 0.2241 | 0.1674 | 12.19 | 0.0397* |
| AD | 0.8537 | 1 | 0.8537 | 0.3267 | 46.44 | 0.0065* |
| Curvature | 0.0887 | 1 | 0.0887 | -0.2016 | 4.83 | 0.1155 |
| Residual | 0.0551 | 3 | 0.0184 |  |  |  |
| Lack of Fit | 0.0405 | 1 | 0.0405 |  | 5.51 | 0.1434 |
| Pure Error | 0.0147 | 2 | 0.0073 |  |  |  |
| R^2^ | 0.9659 |  |  |  |  |  |
| R^2^ adjusted | 0.8977 |  |  |  |  |  |

**(d) Response 4: DPPH**

| **Source** | **Antioxidant activity determination (DPPH )** | | | | | |
| --- | --- | --- | --- | --- | --- | --- |
|  | **Sum of Squares** | **df** | **Mean Square** | **Coefficient estimate** | **F-value** | **p-value** |
| Model | 1.124E+05 | 7 | 16056.74 |  | 17021.28 | < 0.0001* |
| A-Solvent ratio | 29238.46 | 1 | 29238.46 | 60.46 | 30994.83 | < 0.0001* |
| B-Temperature | 22029.01 | 1 | 22029.01 | 52.47 | 23352.30 | < 0.0001* |
| C-Time | 3282.12 | 1 | 3282.12 | -20.26 | 3479.28 | 0.0003* |
| D-Solid/Solvent | 48105.82 | 1 | 48105.82 | 77.55 | 50995.56 | < 0.0001* |
| AB | 1751.73 | 1 | 1751.73 | 14.80 | 1856.96 | 0.0005* |
| AC | 70.92 | 1 | 70.92 | -2.98 | 75.18 | 0.0130* |
| AD | 7919.11 | 1 | 7919.11 | 31.46 | 8394.82 | 0.0001* |
| Curvature | 19344.57 | 1 | 19344.57 | -94.16 | 20506.61 | < 0.0001 |
| Pure Error | 1.89 | 2 | 0.9433 |  |  |  |
| R^2^ | 0.9996 |  |  |  |  |  |
| R^2^ adjusted | 0.9989 |  |  |  |  |  |

**Table S3: ANOVA and regression analysis of CCRD parameters responses of Study 3**

**(a) Response 1: Total carotenoids (ANOVA for Reduced Linear model)**

| **Total carotenoids content** | | | | | | |
| --- | --- | --- | --- | --- | --- | --- |
| **Source** | **Sum of Squares** | **df** | **Mean Square** | **F-value** | **p-value** |  |
| **Model** | 1.05 | 4 | 0.2629 | 4.65 | 0.0135* | significant |
| A-Temperature | 0.1558 | 1 | 0.1558 | 2.75 | 0.1193 |  |
| B-Time | 0.0031 | 1 | 0.0031 | 0.0540 | 0.8196 |  |
| C-Solvent: solid | 0.6805 | 1 | 0.6805 | 12.03 | 0.0038* | significant |
| AB | 0.2122 | 1 | 0.2122 | 3.75 | 0.0733 |  |
| **Residual** | 0.7922 | 14 | 0.0566 |  |  |  |
| Lack of Fit | 0.5879 | 10 | 0.0588 | 1.15 | 0.4846 | not significant |
| Pure Error | 0.2043 | 4 | 0.0511 |  |  |  |
| **Cor Total** | 1.84 | 18 |  |  |  |  |
| R^2^ | 0.6973 |  |  |  |  |  |
| R^2^ adjusted | 0.5476 |  |  |  |  |  |

**(b) Response 2: TPC (ANOVA for Reduced Quadratic model)**

| **TPC** | | | | | | |
| --- | --- | --- | --- | --- | --- | --- |
| **Source** | **Sum of Squares** | **df** | **Mean Square** | **F-value** | **p-value** |  |
| **Model** | 2.06 | 5 | 0.4124 | 48.05 | < 0.0001 | significant |
| A-Temperature | 0.0006 | 1 | 0.0006 | 0.0702 | 0.7951 |  |
| B-Time | 0.1008 | 1 | 0.1008 | 11.75 | 0.0045 |  |
| C-solvent:solid | 1.09 | 1 | 1.09 | 126.70 | < 0.0001 |  |
| A² | 0.8699 | 1 | 0.8699 | 101.33 | < 0.0001 |  |
| C² | 0.0311 | 1 | 0.0311 | 3.62 | 0.0796 |  |
| **Residual** | 0.1116 | 13 | 0.0086 |  |  |  |
| Lack of Fit | 0.1025 | 9 | 0.0114 | 5.01 | 0.0676 | not significant |
| Pure Error | 0.0091 | 4 | 0.0023 |  |  |  |
| **Cor Total** | 2.17 | 18 |  |  |  |  |
| R^2^ | 0.9487 |  |  |  |  |  |
| R^2^ adjusted | 0.9289 |  |  |  |  |  |

**(c) Response 3: FRAP (ANOVA for Reduced Quadratic model)**

| **FRAP** | | | | | | |
| --- | --- | --- | --- | --- | --- | --- |
| **Source** | **Sum of Squares** | **df** | **Mean Square** | **F-value** | **p-value** |  |
| **Model** | 2.15 | 6 | 0.3576 | 16.70 | < 0.0001 | significant |
| A-Temperature | 0.0028 | 1 | 0.0028 | 0.1319 | 0.7228 |  |
| 9B-Time | 0.0163 | 1 | 0.0163 | 0.7602 | 0.4004 |  |
| C-solvent:solid | 1.66 | 1 | 1.66 | 77.40 | < 0.0001 |  |
| AB | 0.0788 | 1 | 0.0788 | 3.68 | 0.0792 |  |
| B² | 0.2258 | 1 | 0.2258 | 10.55 | 0.0070 |  |
| C² | 0.1168 | 1 | 0.1168 | 5.46 | 0.0377 |  |
| **Residual** | 0.2569 | 12 | 0.0214 |  |  |  |
| Lack of Fit | 0.2318 | 8 | 0.0290 | 4.61 | 0.0782 | not significant |
| Pure Error | 0.0251 | 4 | 0.0063 |  |  |  |
| **Cor Total** | 2.40 | 18 |  |  |  |  |
| R^2^ | 0.8931 |  |  |  |  |  |
| R^2^ adjusted | 0.8396 |  |  |  |  |  |

**(d) Response 4: ABTS (ANOVA for Reduced Quadratic model)**

| **ABTS** | | | | | | |
| --- | --- | --- | --- | --- | --- | --- |
| **Source** | **Sum of Squares** | **df** | **Mean Square** | **F-value** | **p-value** |  |
| **Model** | 2.06 | 6 | 0.3437 | 49.99 | < 0.0001 | significant |
| A-Temperature | 0.0016 | 1 | 0.0016 | 0.2343 | 0.6371 |  |
| B-Time | 0.0007 | 1 | 0.0007 | 0.0978 | 0.7598 |  |
| C-solvent:solid | 1.70 | 1 | 1.70 | 246.60 | < 0.0001 |  |
| AB | 0.0586 | 1 | 0.0586 | 8.52 | 0.0129 |  |
| A² | 0.1497 | 1 | 0.1497 | 21.78 | 0.0005 |  |
| C² | 0.1949 | 1 | 0.1949 | 28.35 | 0.0002 |  |
| **Residual** | 0.0825 | 12 | 0.0069 |  |  |  |
| Lack of Fit | 0.0817 | 8 | 0.0102 | 50.88 | 0.0009 | significant |
| Pure Error | 0.0008 | 4 | 0.0002 |  |  |  |
| **Cor Total** | 2.14 | 18 |  |  |  |  |
| R^2^ | 0.9615 |  |  |  |  |  |
| R^2^ adjusted | 0.9423 |  |  |  |  |  |

**(e) Response 5: DPPH (ANOVA for Reduced Quadratic model)**

| **DPPH** | | | | | | |
| --- | --- | --- | --- | --- | --- | --- |
| **Source** | **Sum of Squares** | **df** | **Mean Square** | **F-value** | **p-value** |  |
| **Model** | 6.86 | 7 | 0.9801 | 16.22 | < 0.0001 | significant |
| A-Temperature | 0.0085 | 1 | 0.0085 | 0.1409 | 0.7146 |  |
| B-Time | 0.0002 | 1 | 0.0002 | 0.0037 | 0.9525 |  |
| C-solvent:solid | 1.11 | 1 | 1.11 | 18.40 | 0.0013 |  |
| AB | 0.4460 | 1 | 0.4460 | 7.38 | 0.0200 |  |
| A² | 2.56 | 1 | 2.56 | 42.30 | < 0.0001 |  |
| B² | 0.4835 | 1 | 0.4835 | 8.00 | 0.0164 |  |
| C² | 2.11 | 1 | 2.11 | 34.87 | 0.0001 |  |
| **Residual** | 0.6646 | 11 | 0.0604 |  |  |  |
| Lack of Fit | 0.5788 | 7 | 0.0827 | 3.85 | 0.1051 | not significant |
| Pure Error | 0.0858 | 4 | 0.0215 |  |  |  |
| **Cor Total** | 7.53 | 18 |  |  |  |  |
| R^2^ | 0.9117 |  |  |  |  |  |
| R^2^ adjusted | 0.8555 |  |  |  |  |  |

**Table S4: Elemental composition of unprocessed and Microwave treated carrot rejects after the extraction of bioactive components**

| **Carrot samples** | **Carbon (%)** | **Hydrogen (%)** | **Nitrogen (%)** | **Oxygen (%)** |
| --- | --- | --- | --- | --- |
|  |  |  |  |  |
| Untreated | 40.2 ±0.07 | 6.26 ± 0.039 | 0.94 ±0.03 | 52.60 ±0.02 |
| MAE | 46.5 ±0.05 | 6.46 ± 0.41 | 0.97 ±0.13 | 46.07 ±0.03 |
